# Time-Dependent Transcriptional Changes in Axenic *Giardia duodenalis* Trophozoites

**DOI:** 10.1371/journal.pntd.0004261

**Published:** 2015-12-04

**Authors:** Brendan R. E. Ansell, Malcolm J. McConville, Louise Baker, Pasi K. Korhonen, Neil D. Young, Ross S. Hall, Cristian A. A. Rojas, Staffan G. Svärd, Robin B. Gasser, Aaron R. Jex

**Affiliations:** 1 Faculty of Veterinary and Agricultural Sciences, University of Melbourne, Parkville, Victoria, Australia; 2 Bio21 Molecular Science and Biotechnology Institute, University of Melbourne, Parkville, Victoria, Australia; 3 Department of Cell & Molecular Biology, Biomedical Center, Uppsala University, Uppsala, Sweden; University of Zurich, SWITZERLAND

## Abstract

*Giardia duodenalis* is the most common gastrointestinal protozoan parasite of humans and a significant contributor to the global burden of both diarrheal disease and post-infectious chronic disorders. Although *G*. *duodenalis* can be cultured axenically, significant gaps exist in our understanding of the molecular biology and metabolism of this pathogen. The present study employed RNA sequencing to characterize the mRNA transcriptome of *G*. *duodenalis* trophozoites in axenic culture, at log (48 h of growth), stationary (60 h), and declining (96 h) growth phases. Using ~400-times coverage of the transcriptome, we identified 754 differentially transcribed genes (DTGs), mainly representing two large DTG groups: 438 that were down-regulated in the declining phase relative to log and stationary phases, and 281 that were up-regulated. Differential transcription of prominent antioxidant and glycolytic enzymes implicated oxygen tension as a key factor influencing the transcriptional program of axenic trophozoites. Systematic bioinformatic characterization of numerous DTGs encoding hypothetical proteins of unknown function was achieved using structural homology searching. This powerful approach greatly informed the differential transcription analysis and revealed putative novel antioxidant-coding genes, and the presence of a near-complete two-component-like signaling system that may link cytosolic redox or metabolite sensing to the observed transcriptional changes. Motif searching applied to promoter regions of the two large DTG groups identified different putative transcription factor-binding motifs that may underpin global transcriptional regulation. This study provides new insights into the drivers and potential mediators of transcriptional variation in axenic *G*. *duodenalis* and provides context for static transcriptional studies.

## Author Summary

*Giardia* is the most common gastrointestinal protozoan parasite of humans. This parasite causes diarrheal disease and is correlated with post-infectious conditions such as irritable bowel syndrome. In the absence of a vaccine, treatment is limited to drugs such as metronidazole, against which clinical resistance is reported. Effective control of *Giardia* requires a detailed understanding of its biology, and in turn, complete characterization of the standard *in vitro* culture system. Using RNA sequencing assisted by informatics to functionally annotate hypothetical proteins, we investigated transcriptional changes in axenic *Giardia* trophozoites at three growth phases over 96 hours. We found two large groups of differentially transcribed genes that indicate changes in the antioxidant system and central carbon metabolism over time. A putative novel signaling pathway may act together with putative transcription factor-binding motifs to regulate these transcriptional changes. Our results suggest that dissolved oxygen in *Giardia* culture medium may cause oxidative stress early during *in vitro* growth and that oxygen depletion may limit the efficiency of glycolysis in the declining phase. This work enhances our understanding of the transcriptional flexibility and metabolism of *Giardia in vitro*.

## Introduction

*Giardia duodenalis* (syn. *G*. *lamblia* or *G*. *intestinalis*) is a gastrointestinal protozoan parasite, and a major cause of chronic infectious diarrhoea in the developed and developing world. *G*. *duodenalis* infects approximately one billion people world-wide, causing 200–300 million reported clinical cases each year [1]. *G*. *duodenalis* is proposed to account for ~15% of cases of childhood diarrhoea in developing countries [2]. High rates of chronic diarrhoea in the first two years of life is significantly associated with physical and cognitive 'stunting,' and predisposes sufferers to a variety of adult-onset metabolic disorders [3]. In particular, infection with *G*. *duodenalis* is associated with post-infectious gastrointestinal disorders such as irritable bowel syndrome, chronic fatigue, and obesity [4,5]. Control of giardiasis depends primarily on chemotherapeutic treatment with one of two major drug classes: nitroheterocyclics (e.g., metronidazole) and benzamidazoles (e.g., mebendazole) [6,7]. Treatment failure rates as high as 30% are reported for these compounds [8,9], and *in vitro* resistance to widely used chemotypes is documented in isolates from treatment-refractory patients (reviewed in [8,10]). The recently reported increasing incidence of metronidazole treatment-failure in travellers returning to the United Kingdom [11], and toxicity associated with most nitroheterocyclics [6], highlight the need for continued development of anti-giardial drugs. This in turn requires a thorough understanding of the molecular biology of the parasite. Aside from its medical importance, *G*. *duodenalis* is thought to belong to one of the earliest eukaryotic lineages, and therefore serves as a useful model for studies of eukaryotic features such as secretory and organellar protein trafficking [12], cellular differentiation [13,14] and RNA interference [15–17].

*G*. *duodenalis* can be cultured in complex, host cell-free media, which is a rarity among parasitic protists and of great advantage for conducting molecular research. Axenic culture provides an excellent system in which to explore the biology of *G*. *duodenalis* over time and in response to external stimuli, drug perturbation [18–21] and other stressors [22,23]. System-level transcriptomic investigations based on microarray or serial analysis of gene expression, have established that *G*. *duodenalis* trophozoites exhibit clear transcriptional responses to encystation medium [13], protein folding stress [23] and the presence of intestinal epithelial cells [24,25]. More recently, RNA sequencing (RNA-seq) has been used to identify transcriptional start-sites [26], 3’ un-translated regions and polyadenylation variants in mRNA [27], and to compare transcription between different *G*. *duodenalis* assemblages [27]. RNA-seq has also recently been applied to investigate the transcriptional response to oxidative stress in trophozoites [28], and to ultraviolet irradiation in trophozoites and cysts [29]. However, considering the complex nature of the standard culture medium (TYI-S33) for *G*. *duodenalis* trophozoites, and variation between laboratories in how this medium is prepared, comparing studies is challenging, particularly given that each study represents a static time-point observation. Understanding how transcription varies over time in TYI-S33 medium is important to provide context to single time-point studies. In terms of the axenic growth of the parasite, such research can also provide insight into the changes in the metabolic behaviour and demands of *G*. *duodenalis* during different growth phases.

A major challenge for genomic investigations of divergent organisms such as *G*. *duodenalis* relates to the vast numbers of functionally un-annotated gene products. Indeed, around 50% of the proteins predicted in this protist lack functional information. In lieu of *in vitro* characterization, computational protein structure-prediction approaches can provide substantial insight into the putative function of hypothetical proteins [30]. Here, we used RNA-seq coupled with structural homology-based protein annotation, to investigate the longitudinal transcriptional behavior of *G*. *duodenalis* assemblage A (WB isolate) trophozoites under standard laboratory conditions in TYI-S33 medium. This represents the first high-resolution, longitudinal transcriptional data set for this protist. We hypothesize that differential transcription will be evident between log, stationary, and declining growth phases, and that these changes will reflect the metabolic preferences of *G*. *duodenalis* and the pressures of resource exhaustion.

## Materials and Methods

### Trophozoite culture and growth time-point sampling

*Giardia duodenalis* trophozoites (assemblage A, strain WB-1B; [31]) were generously provided by Drs Jaqueline Upcroft and Peter Upcroft and maintained in axenic culture in filtered, complete modified TYI-S33 medium in close-capped t25 flasks (Falcon) according to standard protocols [32]. The growth kinetics of attached trophozoites was charted over 96 hours (h; S1 Fig), from which the following growth phases were estimated: lag (0–24 h), log (24–60 h), stationary (60–72 h) and declining (72–96 h). As attached and suspended populations exhibited generally similar growth dynamics, we focused on the attached population in order to enrich samples for viable trophozoites and minimize the risk of contamination with degraded mRNA from dead cells. In our hands the generation time of WB1B was 5.4 ±1.2 h during log phase. Samples for sequencing were generated on four different weeks as follows. Nine t25 flasks (64 mL total capacity) were filled with 56 mL of medium, inoculated with 10^5^ trophozoites from confluent t25 flasks, and incubated at 37°C. At 48, 60 and 96 h after inoculation, three flasks were selected at random, and supernatant and suspended cells were discarded and replaced with ice-cold phosphate-buffered saline (PBS). Flasks were incubated on ice for no more than 5 minutes to ensure detachment of trophozoites, and the suspensions were transferred to 50 mL falcon tubes and pelleted at 680 x *g* for 5 min at 4°C. Supernatants were discarded, and pellets were combined through re-suspension in 1 mL of PBS before transfer to a 1.5 mL Eppendorf tube. The suspension was pelleted (770 x *g*, 2 min, 22–24°C), re-suspended in 1 mL of TriPure reagent (Roche), and stored at -80°C.

### RNA extraction, library preparation and sequencing

RNA was extracted from TriPure reagent according to the manufacturer’s instructions within four weeks of sample preparation. The dried RNA pellet was re-suspended in reverse-osmosis deionized water (H_2_O) and treated with Turbo DNAse (Ambion) according to the manufacturer’s instructions. The DNAse-treated RNA was electrophoresed, and large and small subunits of nuclear rRNA bands were examined as a proxy for RNA integrity. RNA concentration was estimated by fluorometry (Qubit) and further quality control was performed using a BioAnalyzer (Agilent). Polyadenylated RNA was purified from 10 μg of total RNA using Sera-mag oligo(dT) beads, fragmented to a length of 100–500 bases, reverse transcribed using random hexamers, end-repaired, and adaptor-ligated, according to the manufacturer's instructions (Illumina). Ligated products (~300 bp) were excised from agarose and PCR-amplified. Products were purified over a MinElute column (Qiagen) and paired-end sequenced (100 bp; non-normalised cDNA) using the Ilumina HiSeq 2000 (Yourgene Biosciences, Taiwan).

### Bioinformatic processing

Adapters were trimmed from raw reads using Trimmomatic [33] (sliding window: 4 bp, minimum average PHRED quality: 20; leading and trailing: 3 bp; minimum read length: 40 bp), and overlapping read pairs were merged using SeqPrep (downloaded 2 June 2014 https://github.com/jstjohn/SeqPrep) with default parameters. The merged reads were combined with unpaired and non-overlapping paired reads from Trimmomatic output, and all were mapped as single-ended reads to the accepted *G*. *duodenalis* gene models (assemblage A genome, WB strain, release 3.1; GiardiaDB.org; [27][34]), using RSEM [35]. Transcripts-per-million (TPM) for each gene were averaged across replicates from each growth phase, and used to rank genes according to relative transcriptional abundance. Expected counts for each gene were submitted to EBSeq [36], incorporating median normalization, and DTGs were identified using a false discovery rate (FDR) of <0.05. Fold-change in transcription between growth phases was calculated using the normalized mean expected counts output from EBSeq. As a further filter, only those genes with at least 10 mean expected counts in at least one growth phase were included in further analysis.

### Data analysis

Feature detection was calculated as a function of mapped read depth, using the counts module in QualiMap (v1.0) with the–k 10 flag (denoting a minimum mapped read threshold of 10) [37]. Saturation plots were displayed in Excel (Microsoft). Heat maps were generated in R (v3.0.2) using the heatmap module. KEGG BRITE terms associated with peptides in the Kyoto Encyclopedia of Genes and Genomes (KEGG) database (release 69.0), were transferred to the closest homolog in *G*. *duodenalis* using BLASTp (lower expect threshold of 10^−5^; [38]). Gene ontology (GO) terms for the predicted *G*. *duodenalis* proteome were retrieved from GiardiaDB.org; and sensitive structure-based homology searches were performed for DTGs annotated as ‘hypothetical’ or ‘deprecated,’ and for peptides encoded by highly transcribed (top 100) genes, using I-TASSER software (v3.0; [30,39]). Briefly, I-TASSER generates putative three-dimensional structural models from amino acid sequences, incorporating predicted secondary structure and the consensus model from multiple threading programs, followed by iterative molecular dynamics simulation to minimize free energy [30]. The closest structural homolog available in the Research Collaboratory for Structural Bioinformatics Protein Data Bank (RCSB PDB; rcsb.org), and consensus GO terms associated with the ten best structural matches, are then inferred for the putative model. Bar charts and box plots describing transcriptional abundance were generated using Excel (Microsoft) and Prism software (GraphPad). For bar charts, mean normalized expected counts are plotted with standard error derived from pre-normalised expected counts for biological quadruplicates. Putative transcription factor (TF)-binding motifs in the promoter regions of DTGs were identified within 400 bp upstream of the start codon in non-overlapping (i.e., non-coding) regions using DREME [40]; cf [41]. Promoters from a DTG group of interest were interrogated using promoters from another DTG group as the background. Interacting TFs for homologous motifs in yeast (all available databases) were predicted using TOMTOM [42]. For each motif, the density of the 5’ nucleotide position (both forward, and reverse complement) was calculated as a function of promoter length, and displayed together with a histogram of the corresponding promoter lengths using R.

### Statistical analysis

The coefficient of variation (SD ÷ average; denoting variation in transcript abundance between biological replicates) was calculated for all transcribed genes encoding variant-specific surface proteins (VSPs). Pearson correlation was used to compare transcriptional abundance (FPKM and RPKM) values from independently generated transcriptomic data sets. Fisher exact tests were used to determine significantly over-represented (i.e., enriched) KEGG BRITE terms within DTG groups. GO enrichment analysis was performed using the BinGO module in Cytoscape [43,44] by firstly providing a background of GO terms specific to *G*. *duodenalis*, incorporating all GO terms from GiardiaDB.org and those terms from I-TASSER with confidence scores ≥0.3. The enriched Biological Process GO terms within DTG groups were then identified using Fisher exact testing (FDR < 0.05). To further minimize false positives in this analysis, resultant GO terms with fewer than ten associated genes in the background, were discarded. Fisher permutation tests were also used to compare the average transcription level of genes of interest between growth phases.

### Accession numbers

Open reading frames for genes in this article, quoted according to GiardiaDB.org: GL50803_87577; GL50803_7195; GL50803_10403; GL50803_33769; GL50803_27266; GL50803_23756; GL50803_16568; GL50803_27266.

## Results

**Fig 1:**
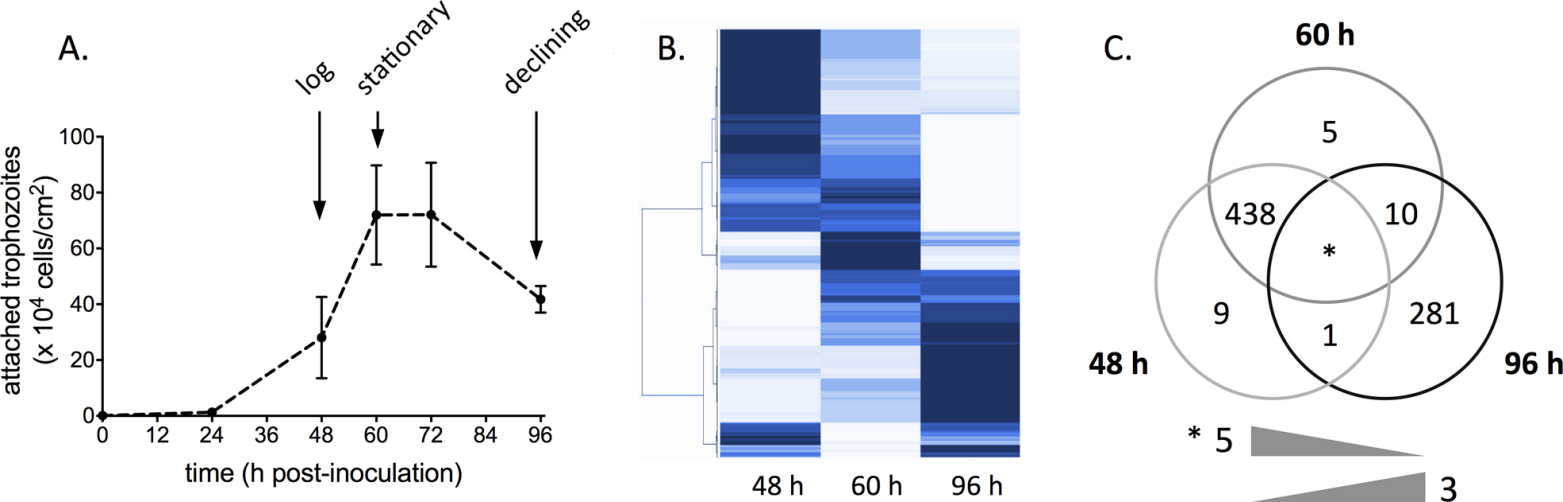
Trophozoite growth profile and differential gene transcription. A: Average density of attached *Giardia duodenalis* trophozoites during *in vitro* growth. Error bars represent ± 1 SEM. B: Heat map displaying transcriptional abundance of all transcribed genes at successive growth phases. Light and dark blue indicate high and low transcriptional abundance respectively. C: Differentially transcribed genes at log, stationary, and declining phases of *in vitro* growth. Numbers represent up-regulated genes relative to other phase(s). *Five and three genes were significantly up- and down-regulated respectively at successive phases.

We compared the transcriptomes of the attached population of *G*. *duodenalis* trophozoites harvested at 48, 60 and 96 h during axenic culture. These time points corresponded to log phase (~ 28 x 10^4^ attached cells/cm^2^), stationary (confluent monolayer on the flask wall; 72 x 10^4^ cells/cm^2^), and declining phases (detaching; 42 x 10^4^ cells/cm^2^) respectively (Figs 1A and S1). In total, 473.76 million RNA-seq reads were produced. For each of 12 samples, an average of 25.25 million high-quality reads (i.e. 92% of all filtered reads) were mapped to the *G*. *duodenalis* gene models (WB strain [27,34]). The average coverage depth was 405-times (S1 Table), and novel transcript detection as a function of the number of mapped reads was saturated for all samples (S2 Fig). After filtering, transcripts were detected from 7,637 ORFs (78.9% of all predicted ORFs), of which 2,347 had functional annotations (98.8% of all annotated), 3,208 were hypothetical (90.5% of all hypothetical), and 2,082 were deprecated (55.3% of all deprecated). When the relative transcriptional abundance (TPM) for genes at each growth phase was compared, values ranged across four orders of magnitude (S3 Fig). The five most highly transcribed genes across the three phases were a translation elongation factor (EF1-beta), ornithine carbamoyltransferase, glyceraldehyde-3-phosphate dehydrogenase, and two ribosomal proteins (S15A and P2C). Among the top 100 most highly transcribed genes in each phase were arginine deiminase, carbamate kinase, enolase, variant-specific surface proteins (VSPs), peroxiredoxin-1ai, two putative thioredoxins and two protein disulfide isomerases (S2 Table). The normalized mean expected counts for genes at each growth phase were displayed in a heat map, and a progressive trend of up- or down-regulation was identified for the majority of the detected genes (Fig 1B). During the axenic growth and decline of *G*. *duodenalis* trophozoites over 96 hours, 754 genes were differentially transcribed at statistical significance (S3 Table); including 438 genes (47% hypothetical; 6% deprecated) down-regulated at the declining phase relative to the log and stationary phases; and 281 genes (49% hypothetical; 19% deprecated) up-regulated at the declining phase relative to both of the earlier phases. On average, fewer than ten genes were differentially transcribed in other comparisons (Fig 1C), and thus statistical analyses were restricted to the two large DTG groups, hereafter termed ‘down-regulated’ and ‘up-regulated’ in the declining phase.

The median fold change for differentially transcribed down- and up-regulated gene groups was 1.85 (IQR = 0.77) and 2.12 (IQR = 0.8) respectively. Using I-TASSER [30], putative structures could be generated for 224 of 234 (95.2%) hypothetical and deprecated proteins within the down-regulated group, and 141 of 190 (74.2%) such proteins in the up-regulated group. The lower availability of structural models for the up-regulated group is due to the presence of 31 genes (16.3%) encoding peptides of >1500 amino acids, which are too large for analysis in the I-TASSER software [30].

### Gene functions down-regulated during axenic growth

GO enrichment analysis revealed significant over-representation of 113 ‘Biological Process’ terms in the down-regulated gene group, of which 49 were unique to this group, including ‘energy derivation by oxidation of organic compounds,’ ‘signaling,’ and ‘locomotion’ (S3 Table). Enriched KEGG BRITE annotations included the NEK kinase family, threonine peptidases, ubiquitin conjugating enzymes (E2) and the T1 proteasome family (S4 Table). The down-regulated genes annotated with oxidoreductase activity (GO:0055114 and/or GO:0016491) included glutamate synthase, 6-phosphogluconate dehydrogenase, alcohol dehydrogenase and nitroreductase-1 as well as a number of hypothetical proteins with predicted structural similarity to glutamate synthase, hydroxylamine oxidoreductase, thiol-cycling enzymes (protein disulfide isomerase and thiol:disulfide protein dsbA), a ferretin-like Dps-like peroxide resistance protein and a nickel-binding superoxide dismutase (Table 1). Further investigation of down-regulated genes associated with ubiquitin-conjugating and protease activity, revealed four ubiquitinylating enzymes (two paralogs of a 28.4 kDa E2, a 17 kDa E2 and the ubiquitin ligase UBC3), and four beta-subunits of the 20S proteasome (S4 Fig).

**Table 1:** Down-regulated genes with predicted oxidoreductase activity (GO:0055114 'oxidation reduction' and/or GO:0016491 'oxidoreductase activity').

		Closest structural homolog in the Protein Data Bank						
Accession no. (GL50803)	Genome annotation	Species	Protein name	PDB code	TM score	RMSD (Å)	% AA identity	% Coverage
14759	6-phosphogluconate dehydrogenase, decarboxylating							
13350	Alcohol dehydrogenase lateral transfer candidate							
7260	Aldose reductase							
15460	Dynein-like protein							
7195	Glutamate synthase*	*Methylophilus methylotrophus*	Trimethylamine dehydrogenase	1DJQ	0.7084	1.41	10.0	71.86
6175	Nitroreductase 1**							
88581	Synaptic glycoprotein SC2							
9068	Hypothetical protein	*Thermus thermophilus*	Cytochrome c oxidase polypeptide i+iii	2YEV	0.7059	2.72	12.1	90.91
15154	Hypothetical protein	*Streptococcus pyogenes*	Dps-like peroxide resistance protein	2WLA	0.6592	2.42	11.4	75.12
4690	Hypothetical protein	*Legionella pneumophila*	Effector protein B	4JW1	0.8506	1.61	7.5	91.62
87577	Hypothetical protein	*Azospirillum brasilense*	Glutamate synthase [NADPH] small chain	2VDC	0.9178	2.07	14.1	98.68
15445	Hypothetical protein	*Megathura crenulata*	Hemocyanin KLH1	4BED	0.8475	2.43	12.8	95.45
17278	Hypothetical protein	*Megathura crenulata*	Hemocyanin KLH1	4BED	0.7172	3.43	5.6	87.54
29796	Hypothetical protein	*Megathura crenulata*	Hemocyanin KLH1	4BED	0.7377	3.19	8.7	82.69
4692	Hypothetical protein	*Megathura crenulata*	Hemocyanin KLH1	4BED	0.8456	2.56	10.6	98.74
14921	Hypothetical protein	*Candidatus kuenenia stuttgartiensis*	Hydroxylamine oxidoreductase	4N4J	0.8553	2.94	10.3	93.57
27887	Hypothetical protein	*Bacillus subtilis*	Methylthioribose-1-phosphate isomerase	2YVK	0.6671	2.73	9	92.86
15599	Hypothetical protein	*Homo sapiens*	Neuronal acetylcholine receptor subunit α7	2MAW	0.6878	1.68	8.1	75.00
88556	Hypothetical protein	*Homo sapiens*	Otu domain-containing protein 5	3TMP	0.4592	2.11	14.3	49.09
17400	Hypothetical protein	*Saccharomyces cerevisiae*	Pho85 cyclin Pho80	2PK9	0.6544	1.89	13.3	69.11
17375	Hypothetical protein	*Vitis vinifera*	Polyphenol oxidase, chloroplast	2P3X	0.7217	3.04	13.4	82.60
3951	Hypothetical protein	*Saccharomyces cerevisiae*	Protein disulfide-isomerase	3BOA	0.8572	1.98	12.5	92.36
103988	Hypothetical protein	*Neisseria meningitidis*	Putative uncharacterized protein	3VJZ	0.5651	3.83	12.3	82.26
11341	Hypothetical protein	*Vibrio parahaemolyticus*	Sensor protein	3I9Y	0.7838	1.9	7.9	96.20
8048	Hypothetical protein	*Escherichia coli*, *homo sapiens*	Soluble cytochrome b562 and glucagon receptor	4L6R	0.816	2.73	7.7	90.02
3581	Hypothetical protein	*Streptomyces coelicolor*	Superoxide dismutase [Ni]	3G4X	0.7515	1.94	8.8	85.07
5810	Hypothetical protein	*Psychrobacter arcticus*	Uncharacterized protein	2RE7	0.8112	1.92	16.1	94.66
11129	Hypothetical protein	*Homo sapiens*	Uncharacterized protein Kiaa0174	3FRR	0.5589	1.51	12.6	57.78
10244	Deprecated	*Helicobacter pylori*	Cag pathogenicity island protein (Cag18)	3ZCI	0.6398	2.86	4.2	84.43
14932	Deprecated	*Zea mays*	Histidine-containing phosphotransfer protein	1WN0	0.7383	2.39	16.4	87.01
35700	Deprecated	*Listeria monocytogenes*	Lmo 0438 protein	2XL4	0.7886	1.74	6.1	98.00
37288	Deprecated	*Vibrio cholerae*	Thiol:disulfide interchange protein DsbA	2IJY	0.5567	2.92	3.5	82.26

Structural homology information not determined for annotated ORFs.

* Annotated glutamate synthase disambiguated from GL50803_87577 by structural homology searching

** Referred to as NR-2 by Müller et al [86].

### A putative two-component redox-response system in *Giardia duodenalis*

**Fig 2:**
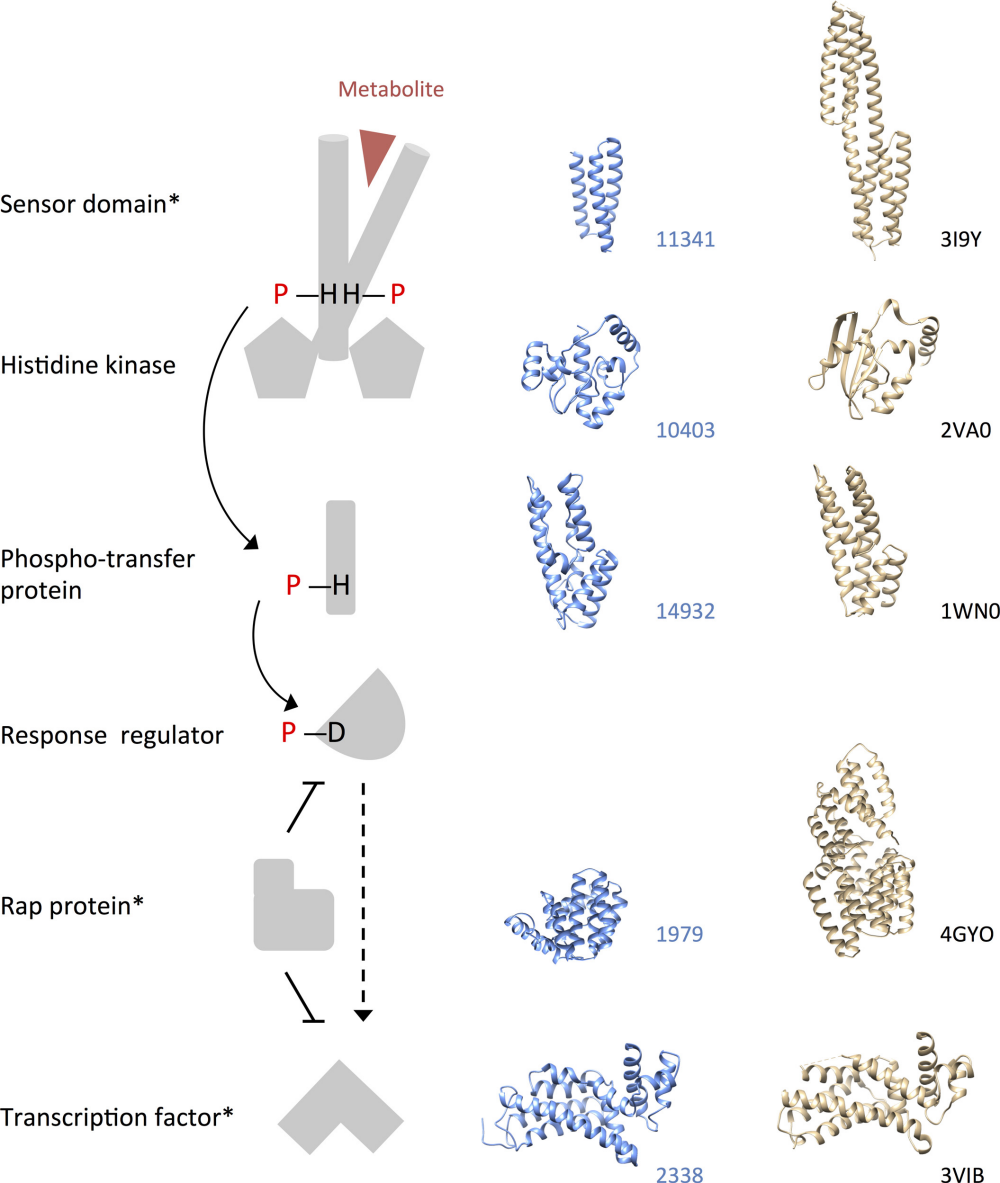
A putative two-component-like signaling pathway in *Giardia duodenalis*. A generalized complete two-component signaling pathway cartoon is displayed in grey. Putative structures for hypothetical *G*. *duodenalis* proteins are displayed in blue, with accession numbers (GL50803) at right. The genes encoding these hypothetical proteins are down-regulated at the declining phase relative to log and stationary phases, with the exception of the histidine kinase. For each putative *G*. *duodenalis* structure, the closest structural match available in the Research Collaboratory for Structural Bioinformatics Protein Data Bank is shown in gold, with the PDB code at right. *single-copy ortholog identified in *G*. *duodenalis* assemblages B and E (GiardiaDB.org).

We identified an annotated and a hypothetical glutamate synthase in the down-regulated gene group. Interrogation of putative structures for each protein revealed that the hypothetical glutamate synthase (GL50803_87577) was most structurally similar to a bacterial glutamate synthase beta sub-unit (PDB code: 2VDC), whereas surprisingly, the annotated glutamate synthase (GL50803_7195) was most similar in structure to trimethylamine (TMA) dehydrogenase from *Methylophilus methylotrophus* (PDB code: 1DJQ). Putative homologs of a TMA sensor protein, a Rap modulator protein (C-terminal domain only), and a phosphotransfer protein were also present in the down-regulated gene group. The former two are present as single-copy orthologs in other *G*. *duodenalis* assemblages (B and E; GiardiaDB.org). These findings suggest the presence of a two-component-like signaling system in *G*. *duodenalis*. Histidine kinases are integral to two-component systems, and further interrogation of putative structures for hypothetical proteins revealed putative histidine kinase activity for GL50803_10403 (Fig 2).

### Gene functions up-regulated in the declining phase

The up-regulated gene group was enriched for 107 ‘Biological Process’ GO terms (43 unique), including ‘gene silencing,’ ‘mitosis,’ and ‘microtubule-based process’ (S3 Table). Enriched KEGG BRITE annotations in this group related to carbon fixation, oxidoreductases and the cytoskeleton (S4 Table). Further investigation of oxidoreductase-related genes in the up-regulated group revealed two pyruvate:ferredoxin oxidoreductase (PFOR) paralogs, and a hypothetical protein with structural homology to a GntR transcription factor (S2 Table).

### Changes in antioxidant and glycolytic enzyme transcription

**Fig 3:**
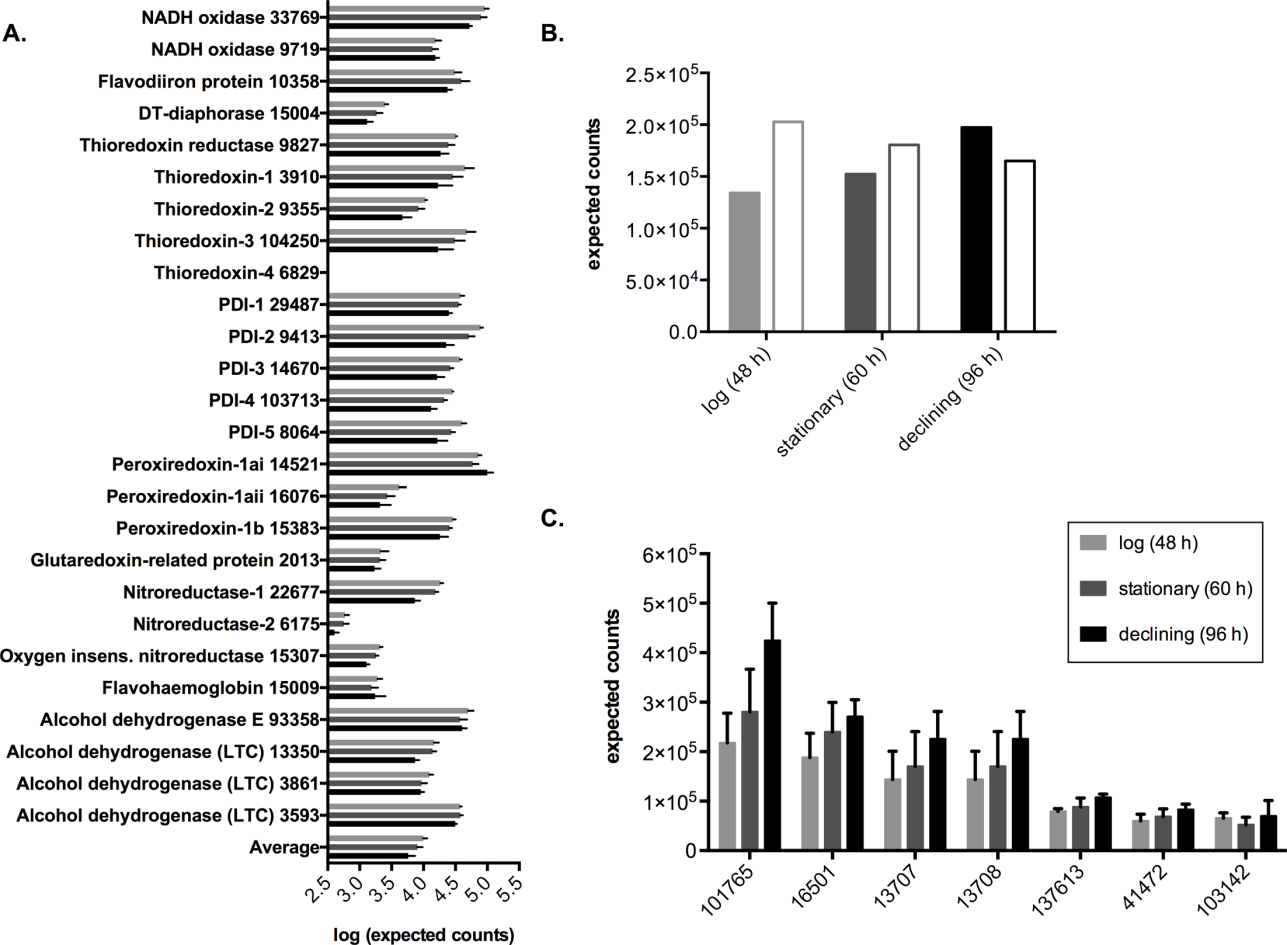
Transcriptional profiles for the antioxidant system and surface proteins during *in vitro* growth. A: Transcriptional abundance for currently annotated antioxidant genes, and average (note the log scale). Gene accession numbers (GL50803) are displayed after the gene description. B: Aggregate transcription for variant-specific surface protein (VSP)-coding genes (filled; expected counts ÷ 10 for display purposes) and high-cysteine membrane protein (HCMP)-coding genes (outline). C: Transcriptional profiles for the seven most highly transcribed VSPs. Mean normalized transcriptional abundance is presented for panels A and C. Error bars represent ±1 SEM, calculated from pre-normalized expected counts for biological quadruplicates. PDI: protein disulfide isomerase; LTC: lateral transfer candidate; insens.: insensitive.

To contextualize the oxidoreductase-related genes that were differentially transcribed between growth phases, we investigated the transcription of other genes involved in the antioxidant system and glycolysis. Progressive but non-significant decreases in transcriptional abundance were identified for thioredoxin reductase, three putative thioredoxins, three peroxidredoxins and five thioredoxin domain-containing protein disulfide isomerases. Similar patterns were observed for transcripts encoding the oxygen-consuming enzymes NADH oxidase (GL50803_33769; [45]) and flavodiiron protein [46,47] (Fig 3A). The collective transcription of annotated antioxidant enzymes was significantly lower in the declining phase compared to earlier phases (Fisher permutation test, 1000 iterations, *p* = 0.037; Fig 3A).

**Fig 4:**
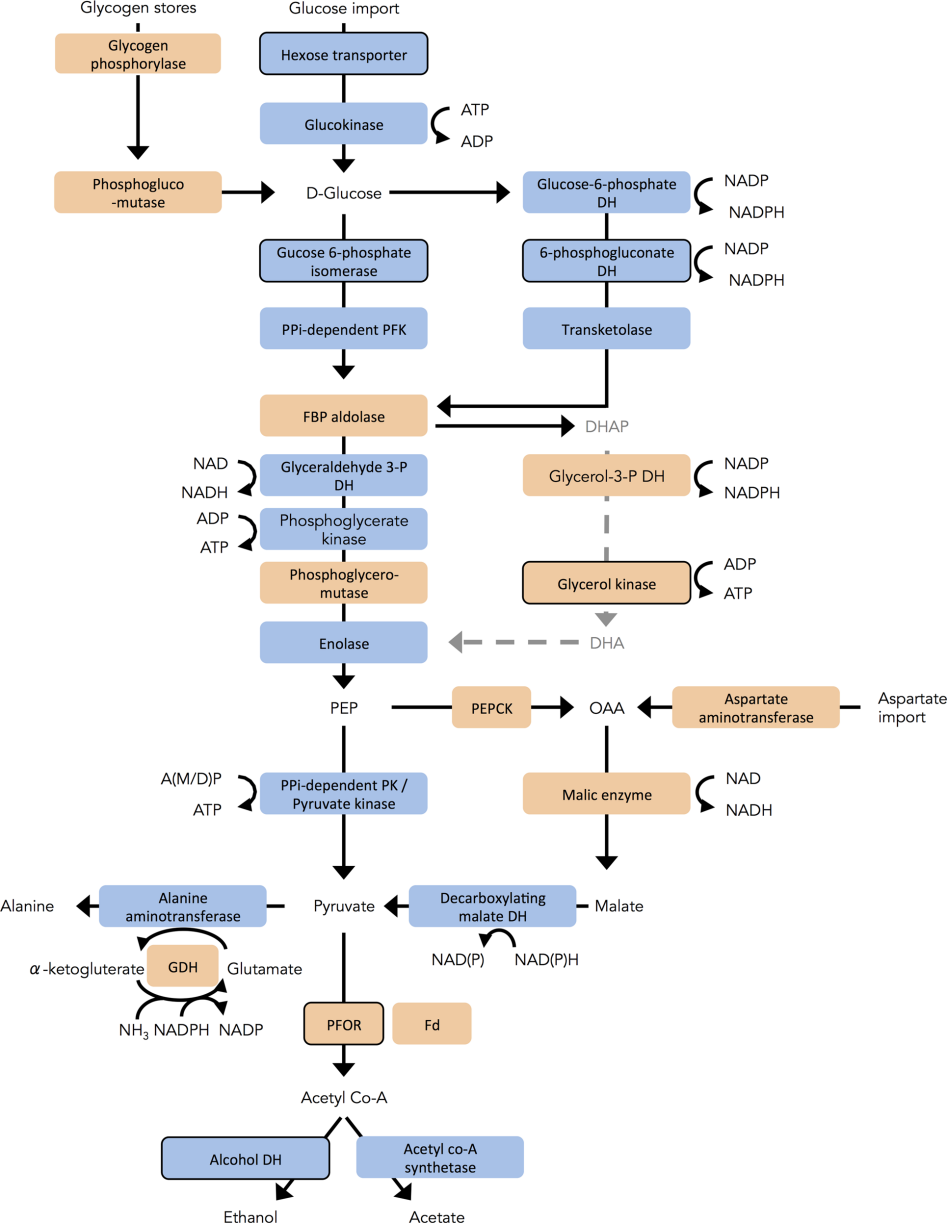
Transcriptional trends for genes encoding glycolytic enzymes. Orange indicates higher transcriptional abundance at the declining phase relative to log phase; and blue indicates the opposite trend. Significant differential transcription is indicated by a dark outline. Only one of three ferredoxin-coding genes (GL50803_27266) exhibits increased transcription in the declining phase. Readers are referred to S5 Table for further information on transcription levels. DH: dehydrogenase; OAA: oxaloacetate; PK: pyruvate kinase; PEP(CK): phosphoenolpyruvate (carboxykinase); DHA(P): dihydroxyacetone (phosphate); A(M/D)P: AMP or ADP; GDH: glutamate dehydrogenase; Fd: ferredoxin.

The presence of genes encoding glycolytic oxidoreductases in both down- and up-regulated gene groups suggested changes in central carbon metabolism during *in vitro* growth. A hexose transporter, and the non-oxidative glycolytic enzyme glucose-6-phosphate isomerase, were also down-regulated over time. Indeed, transcription of the majority of genes representing glycolytic enzymes in both the pentose phosphate and Embden-Meyerhoff pathways, was lowest in the declining phase (Fig 4). Conversely, transcription of a number of glycolytic enzymes downstream of pyruvate increased over time. In addition to significant up-regulation of PFOR-coding genes in the declining phase, genes encoding one of three ferredoxins (GL50803_27266) and a glutamate dehydrogenase also showed greatest transcriptional abundance in this phase (Fig 4).

### Comparison with an/aerobic transcriptomes

Given the robust transcriptional changes in the antioxidant system, we compared the transcriptome for each growth phase with independently generated transcriptomes for WB strain trophozoites cultured under aerobic and anaerobic conditions, as reported by Ma’ayeh et al [28]. When all transcribed genes were considered, our data correlated most closely with the anaerobic transcriptional profile. The transcriptional abundance of annotated antioxidant genes at log and stationary phase, however, correlated best with the aerobic transcriptome, and in the declining phase we observed a shift to stronger correlation with the anaerobic transcriptome (S5 Fig, panels A & B). When glycolytic genes were investigated, there was a trend of increasing correlation with the anaerobic profile over time, and the correlation with the aerobic profile declined after stationary phase. Further dissection of this result revealed separate underlying trends, wherein the genes upstream of pyruvate diverged markedly from the aerobic profile after stationary phase, but little change was seen in genes downstream of pyruvate (S5 Fig, panels C-E).

### Membrane protein transcription during axenic growth

Eleven genes encoding VSPs were identified in the up-regulated group as opposed to only two such genes in the down-regulated group. Further investigation of the transcription levels of 193 transcribed VSPs in our data revealed seven prominently transcribed genes, whereas the rest of the population was relatively lowly transcribed (S6 Fig). Aggregate VSP transcription increased over time, driven by increases in the seven most highly transcribed genes (Fig 3B and 3C), which were consistently highly transcribed in all replicates (S7 Fig). Interestingly, a marked decrease in inter-experimental variation was evident for highly transcribed VSPs in the declining phase relative to earlier phases (S8 Fig). These results prompted investigation of the other major class of membrane proteins in *G*. *duodenalis*, the 60 high-cysteine membrane proteins (HCMPs), whose aggregate transcription in contrast to the VSPs, decreased progressively over time, driven by declining abundance of the most highly transcribed gene quartile (Fig 3B).

### Putative promoter-based transcriptional regulation

**Fig 5:**
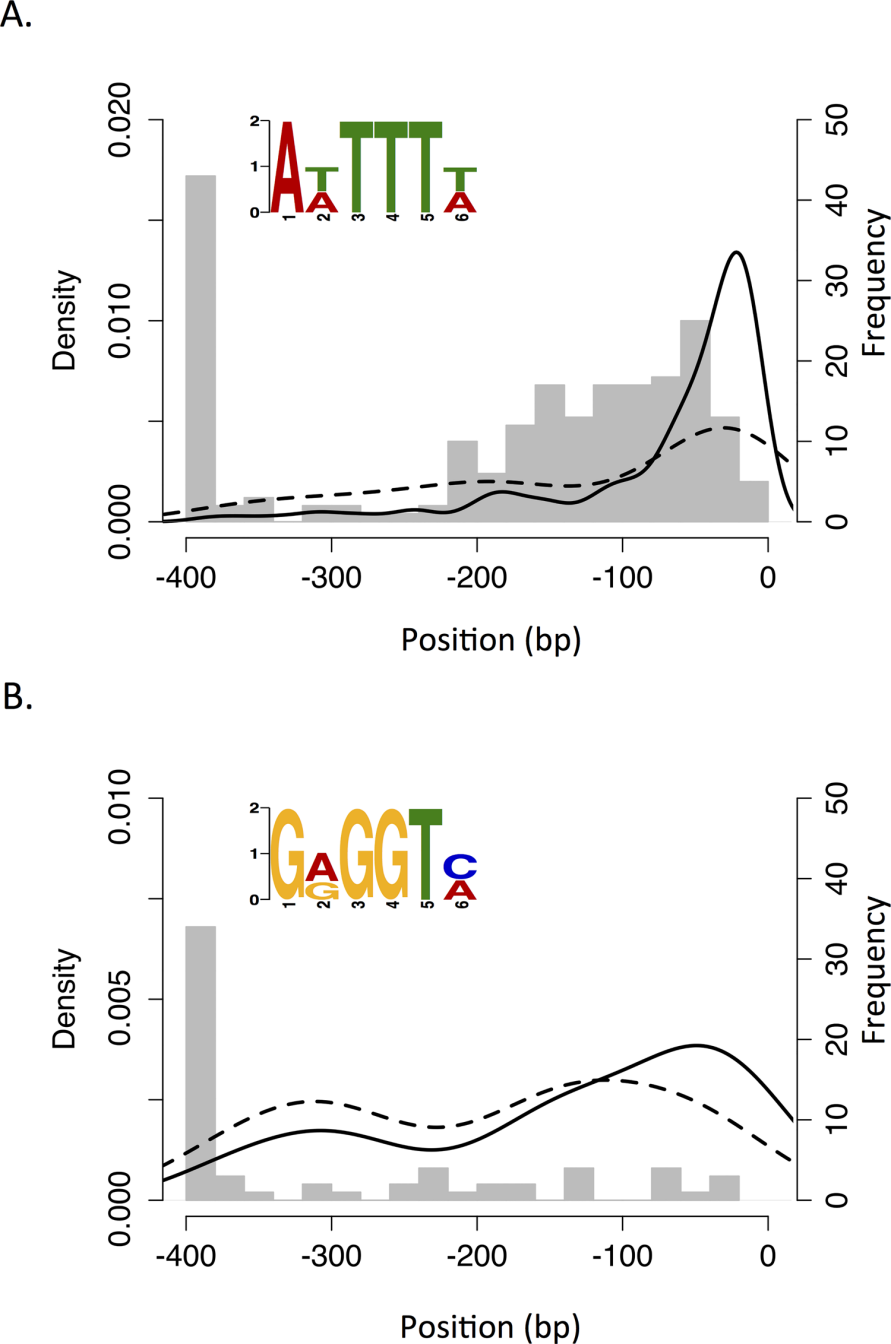
Putative transcription factor-binding promoter motifs in down- and up-regulated differentially transcribed gene groups. A: The AWTTW motif (logo inset) identified in the down-regulated gene group. B: The GRGGTM motif in the up-regulated group. The density of constituent motifs within 400 bp upstream of genes in the DTG group (unbroken line) and in artificially truncated 400 bp promoters (perforated line) is displayed together with a histogram of promoter lengths (grey bars). See Table 2 for motif constituents and statistics.

We hypothesize that DNA-binding transcription factors (TFs) may mediate the dynamic variation in gene transcription evident in *G*. *duodenalis* during axenic growth. The down-regulated gene group did not contain annotated TFs, but did include a putative homolog of the *Neisseria gonorrhoeae* MtrR repressor among three hypothetical protein-coding genes with GO annotations relating to transcriptional regulation (GO:0001071; Fig 2 and S2 Table). The up-regulated group contained an E2F-like TF (GL50803_23756; [48]), a putative TF (GL50803_16568) and four hypothetical proteins annotated with GO:0001071 including the aforementioned GntR homolog (S2 Table). In order to identify putative TF-binding motifs within the promoter regions of genes in the two large DTG groups, we used a similar method to Xu et al. [41] for analysis of the related diplomonad *Spironucleus salmonicida*. Totals of 283 and 178 non-overlapping promoter regions of 8 bp to 400 bp were available for the down- and up-regulated gene groups, respectively. The motif AWTTW was significantly over-represented in the promoters of down-regulated genes relative to promoters of up-regulated genes, and the motif GRGGTM was over-represented in the up-regulated gene promoters in the same way (Table 2). After correction for multiple comparisons, no significant matches to known TF-binding motifs in yeast were found for either motif using TOMTOM. An analysis of the position of each motif within promoter regions revealed robust positional conservation for the AWTTW motif within 100 bp of the start codon, but not for GRGGTM. There was no evidence to suggest that these results are biased by the proportion of non-overlapping genes available for analysis in each group (76% and 72%, respectively); moreover, the motif positional densities remain when only the (artificially truncated) 400 bp promoter regions are considered, indicating little effect of promoter length on motif location (Fig 5).

**Table 2:** Putative transcription factor-binding motifs over-represented in promoters of the down- and up-regulated gene groups.

	Forward	Reverse complement	*p* value	E value
**Down-regulated group**				
**Combined motif**	**AWTTTW**	**WAAAWT**	**4.20E-10**	**3.80E-04**
**Constituents**	AATTTT	AAAATT	6.80E-08	6.30E-02
	ATTTTA	TAAAAT	8.80E-07	8.00E-01
	ATTTTT	AAAAAT	6.90E-04	6.40E+02
	AATTTA	TAAATT	3.60E-03	3.30E+03
**Up-regulated group**				
**Combined motif**	**GRGGTM**	**KACCYC**	**3.00E-12**	**2.20E-06**
**Constituents**	GAGGTC	GACCTC	3.60E-06	2.60E+00
	GAGGTA	TACCTC	3.20E-04	2.40E+02
	GGGGTC	GACCCC	3.90E-03	2.90E+03
	GGGGTA	TACCCC	7.40E-03	5.40E+03

Nucleotide ambiguity codes. W: A or T; R: A or G; M: A or C; K: G or T; Y: C or T.

## Discussion

We studied the transcriptional dynamics of axenic *G*. *duodenalis* trophozoites over the log, stationary, and declining phases (48, 60 and 96 h) of *in vitro* growth. We also generated putative structures for the majority of hypothetical and deprecated proteins encoded by DTGs, gaining insights into the function of divergent gene products that lie beyond reach of conventional sequence-based annotation tools. The majority of genes were transcribed in at least one of the three growth phases investigated, and clear transcriptional variation was evident, implicating effective and directed mechanisms of transcriptional regulation. Strikingly, and contrary to our original hypothesis, trophozoites in non-sparged axenic culture exhibited little difference in transcription between log and stationary phases. Indeed, at both phases, we observed high transcription of a number of genes involved in managing oxidative stress. It should be noted that in order to limit the influence of dead or dying cells on our transcriptomic data, we confined our analyses to adherent cells, and discarded suspended cells prior to sequencing. The suspended population would likely be enriched for both dividing cells and non-viable/dying cells, and it would be of interest to analyze the transcriptomes of these different populations if they could be fractionated. As trophozoites consume oxygen [49,50] and are cultured in a closed system, the pronounced shift in oxidoreductase transcription between log and stationary phases, and the declining phase of axenic growth, is consistent with declining oxygen tension in TYI-S33 medium, and might be associated with a concomitant decline in glucose catabolism and/or entry into a metabolically quiescent state. The present work also found informatic evidence for a two-component–like signaling system that might mediate cytosolic redox or metabolite sensing, and identified different promoter motifs within DTG groups which, together, could contribute to transcriptional regulation.

### Indicators of oxidative stress early during *in vitro* culture

*G*. *duodenalis* features an elaborate antioxidant system that utilizes NAD(P)H to reduce intracellular oxygen and associated reactive oxygen species (ROS). This system both protects iron-containing enzymes, such as PFORs, from oxidative inactivation [51,52] and allows maximal ATP production from glycolysis [53]. As a ‘microaerophile’, *G*. *duodenalis* thrives under dissolved oxygen (dO_2_) concentrations between 5–25 μM, above which dO_2_ becomes cytotoxic [54]. In accordance with the standard trophozoite maintenance protocol [32], we did not sparge dO_2_ from the TYI-S33 medium for this experiment, and medium is expected to contain dO_2_, particularly during the log phase of trophozoite growth. The abundant transcription of genes encoding peroxiredoxin, oxygen-consuming NADH oxidases, thioredoxins and other protein disulfide isomerases is consistent with previous reports [27–29,55]. However, the dynamics of antioxidant transcription in *G*. *duodenalis* under standard culture conditions has not been studied previously. It is likely that the antioxidant system is constitutively highly transcribed to manage transient increases in gut dO_2_ [28,56]. Nevertheless, against this background, we observed down-regulation of the vast majority of antioxidant-coding genes over time, suggesting global regulation of antioxidant transcription.

A number of our results indicate that *G*. *duodenalis* trophozoites may be under oxidative stress at early stages of *in vitro* culture. Firstly, although it was not possible to directly measure dO_2_ without perturbing the standard culture system, correlations with independently generated transcriptional profiles for trophozoites under aerobic and anaerobic conditions [28] can be considered as a proxy measure of dO_2_ tension. At successive growth phases, antioxidant and glycolytic gene transcription shifted from resembling the aerobic transcriptional profile, to the anaerobic profile. The correlation was influenced more strongly by antioxidant transcription than glycolytic enzyme transcription, however glycolytic genes upstream of pyruvate appeared most sensitive to changes in oxygen tension. This strongly suggests that oxygen tension decreases progressively in axenic culture. Furthermore, genes that were down-regulated in our data at the declining phase, encode putative antioxidant proteins, such as an iron-independent superoxide dismutase, and a ferritin-like (iron-sequestering) protein. TYI-S33 medium is supplemented with ammonium ferric citrate, as iron is essential for the activity of enzymes such as PFOR. However iron can also react with dO_2_ to generate ROS. Thus the greater transcriptional abundance of putative iron-sequestering and iron-independent antioxidant proteins at early growth phases, may represent a response to iron- and oxygen-induced oxidative stress. In addition, PFOR enzymes are sensitive to oxidative inactivation [51,52,57], and both PFOR paralogs showed a significant increase in transcription in the declining phase, which is consistent with lower oxygen tension. Lastly, the greater abundance of transcripts encoding elements of the ubiquitin system and proteasomal components at earlier phases, may reflect heightened turn-over of oxidized proteins [58]. However as the proteasome is also important for the demands of protein folding in actively dividing cells, this result requires further investigation.

In the context of global changes in antioxidant transcription, and a suggested decline in dO_2_, we observed inverse transcriptional patterns for the high-cysteine membrane proteins (HCMPs), and variant-specific surface proteins (VSPs), which may indicate differential sensitivity to dO_2_. HCMPs contain disulfide motifs that are common in antioxidant proteins and can both oxidize and reduce substrates such as misfolded proteins, and reduce ROS [59]. HCMPs localize to the nuclear envelope, endoplasmic reticulum, and possibly to the trophozoite plasma membrane [24,59]. The greater aggregate transcription of HCMPs early during *in vitro* growth might relate to protecting membranes from peroxidation [59]. Conversely, aggregate VSP transcription increased over time, which was largely due to progressively greater transcription of seven highly abundant VSP-coding genes, rather than induction of new genes. *G*. *duodenalis* trophozoites transcribe multiple VSP-coding genes, all but one of which are degraded by RNA-interference [17], and thus only a single VSP gene product is displayed on the trophozoite membrane at any time. Our results support previous reports that WB trophozoite populations express relatively few VSPs [60], which may be due to slower VSP turnover in this strain [61]. Specific VSPs are proposed to modulate trophozoite sensitivity to host defenses such as intestinal proteases [62], and VSP suppression is associated with nitroimidazole resistance [19]. Given that the variation in VSP transcription between replicates is particularly low for highly transcribed VSPs in the declining phase, it would be interesting to test whether VSPs are under selection in TYI-S33 medium, or whether the transcriptional increase merely reflects, for example, lower competition from HCMPs for membrane occupancy.

### Glycolysis, oxygen availability & alternative energy sources

The functionally reduced mitochondria, or mitosomes, of *G*. *duodenalis* do not contain enzymes for the tricarboxylic acid cycle or oxidative phosphorylation [34], and this protist is dependent on glycolysis and fermentative metabolism to generate energy from glucose. ATP is generated by direct phosphorylation of AMP and ADP [63]. Electrons liberated during glycolysis are accepted by NAD and NADP, forming NAD(P)H, which must be re-oxidized. Under anaerobic conditions, pyruvate is diverted to ethanol to regenerate NAD, whereas NADP is regenerated through a ‘shunt’ incorporating glutamate dehydrogenase and alanine aminotransferase. Conversely, in the presence of dO_2_, oxidoreductases in the antioxidant system consume NAD(P)H to neutralize dO_2_, ROS, and oxidized biomolecules. Under these conditions, pyruvate is not required for NAD(P) regeneration, and can be further oxidized to acetate. This ‘micro-aerobic’ metabolism maximizes the ATP that is generated from glycolysis.

Here, we observed a trend of decreasing transcriptional abundance for the majority of genes encoding enzymes in the Embden-Meyerhoff and pentose phosphate glycolytic pathways, that convert glucose to pyruvate (Fig 4). TYI-S33 medium contains very high glucose concentrations (55 mM) [32], and thus it is highly unlikely that glucose is exhausted at the declining phase. Instead, the apparent down-regulation of glycolytic pathways may be due to declining dO_2_ availability, which could limit the efficiency of glycolysis. If glycolysis is progressively down-regulated as dO_2_ declines, *G*. *duodenalis* may rely on alternative energy sources. This protist is capable of converting arginine, aspartate and alanine to pyruvate [64,65]. The arginine dihydrolase pathway provides a ready source of ATP in *G*. *duodenalis* and is highly transcribed and stable across all time points studied here. In contrast to glycolysis, which must be coupled to NAD(P)+ regeneration mechanisms, the catabolism of aspartate to pyruvate is effectively redox-neutral in that it does not generate excess NAD(P)H [65]. We observed progressive, but non-significant increases in transcription of two genes involved in aspartate catabolism (Fig 4), which may reflect greater reliance on aspartate for energy as dO_2_ declines. Significant up-regulation of PFORs at the declining phase, and the concomitant down-regulation of alcohol dehydrogenase-coding genes, suggests that a substantial amount of pyruvate is converted to acetate for ATP production even under limited dO_2_ availability (Fig 4). This is supported by reports of acetate production under complete anaerobiasis in *G*. *duodenalis* [64], indicating that pyruvate flux through acetyl-CoA synthase is a resilient mechanism of ATP generation.

The identification of a putative trimethylamine (TMA) dehydrogenase and elements of a putative TMA-NO sensing system (discussed below) in the down-regulated genes, raise the possibility that *G*. *duodenalis* can utilize TMA-NO as a terminal electron acceptor. TMA-NO metabolism has been demonstrated for a variety of gut commensals [66], and the acquisition of key metabolic genes in *G*. *duodenalis* via horizontal gene transfer is well documented [34, 67, 68]. Lastly, the significant up-regulation of glycerol kinase at the stationary and declining phases relative to log phase, could indicate a glycerol-dependent ATP generation pathway in *G*. *duodenalis*. The metabolically similar protist *Entamoeba histolytica* has been shown to divert glycolytic intermediates to glycerol when central glycolytic enzymes are experimentally inactivated, and glycerol kinase is suggested to function in ATP generation [69]. Thus, it would be interesting to investigate the potential of this ‘glycerol shunt’ as an alternative source of ATP in *G*. *duodenalis*.

### Transcriptional regulation

The major shift in transcription between the log and stationary, and the declining growth phases, is likely to involve signaling between cytosolic proteins and nuclear transcription factors. Intriguingly, the observed transcriptional down-regulation of genes related to glycolysis might be mediated by redox-dependent TFs. A putative GntR homolog is up-regulated at the declining phase. In *Corynebacterium glutamicum*, GntR is reported to repress transcription of the gene encoding 6-phosphogluconate dehydrogenase, which is associated with the production of NADPH in the pentose-phosphate pathway. Notably, the gene encoding 6-phosphogluconate dehydrogenase is transcriptionally down-regulated at the declining phase in our data-set, and as mentioned above, transcription of glycolytic genes upstream of pyruvate, such as 6-PGDH, seem to vary more greatly in response to changes in dO_2_ tension (S5 Fig, panel D). Taken together, these findings suggest that declining dO_2_ may inhibit glycolysis and lead to the accumulation of intracellular NAD(P)H. The apparent metabolic shift away from glycolysis may be mediated, at least in part, by redox-sensitive TFs such as the GntR homolog.

Conserved kinases have been classified in *G*. *duodenalis*, many of which localize to the cytoskeleton and might participate in regulation of cell structure or motility. Other kinases, particularly within the massively expanded NEK kinase family, are predicted to lack catalytic activity [70]. Although complete signaling pathways have not been resolved in *G*. *duodenalis*, here we have identified several differentially transcribed structural homologs of proteins that participate in two-component signal transduction. Two-component systems feature histidine kinases and associated sensor domains that detect changes in cytoplasmic or extracellular environmental conditions. Conformational changes in the sensor domain induce histidine autophosphorylation proximal to the kinase domain, and the charged phosphate is subsequently transferred to an effector protein either directly, or via phosphotransferase intermediates. In many cases, the effector translocates to the nucleus to elicit a transcriptional response [71]. Two-component systems are prevalent in bacteria, fungi, plants and free-living protozoa [72], but are little documented in parasitic protozoa and reportedly absent from *Plasmodium falciparum* [73–75]. The putative sensor protein in our data is structurally similar to a transmembrane protein that detects trimethylamine (TMA) in the bacterial periplasm. The predicted *G*. *duodenalis* structure is truncated however, which may indicate a role in intra-membrane or cytosolic chemotaxis. We also identified a histidine kinase homolog, which could represent a kinase class that was previously thought to be absent in *G*. *duodenalis* [70]. Furthermore, the down-regulated gene group included a gene encoding a hypothetical protein with putative structural homology to the MtrR repressor, a TF that is regulated by two-component signaling in *Neisseria gonorrheae* [76] (Fig 2).

Although we did not identify a dimerization domain in the putative histidine kinase or a candidate for the response regulator, the sensor domain, Rap modulator and MtrR homologs are conserved between assemblages B and E of *G*. *duodenalis*, emphasizing the functional importance of these gene products among members of the species complex (Fig 4). Given the absence of two-component systems from metazoans [74], this putative pathway warrants detailed investigation as a possible target for chemotherapeutic intervention.

In further support of coordinated transcriptional regulation, we performed the most comprehensive and sensitive promoter motif search on this species to date, and identified motifs that are enriched in the promoter regions of down- and up-regulated DTG groups. The motif enriched in the down-regulated group (AWTTTW), occurs close to the translation start codon and is likely to be related to the AT-rich transcription initiator motifs reported in previous studies [26,77,78]. At present, little is known about TFs in *G*. *duodenalis* other than those involved in inducing encystation [48,79–82]. It is conceivable that, in the presence of abundant nutrients, transcription is relatively tightly regulated via TF binding to initiator regions. Subsequently, in the declining phase when trophozoites appear to be metabolically stressed, and display physiological changes such as a loss of cytoadherence, less energy might be expended on transcriptional regulation. The GRGGTM motif, which is enriched in the promoters of up-regulated genes, exhibits little positional conservation, perhaps due to the relatively low number of positive promoter sequences (64 *vs* 209 for the AWTTTW motif). Although neither the of the motifs identified in down- and up-regulated genes matched to known TF-binding motifs in yeast, the MtrR and GntR putative TFs identified in these groups are worthy candidates for further investigation, which could link redox/metabolite sensing and transcriptional regulation.

### Conclusions and implications for future studies

By characterizing the dynamics of the mRNA transcriptome of axenically cultured *G*. *duodenalis* trophozoites across growth phases, we identified major changes in gene transcription that relate to central carbon metabolism and the antioxidant system. We also identified a putative signaling pathway and promoter motifs upstream of DTGs that might contribute to transcriptional regulation. We show that transcriptional behaviour of *G*. *duodenalis* trophozoites differs markedly over time in axenic culture, likely reflecting the exploitation and depletion of essential nutrients in a closed culture system. Importantly, the present data indicate that culturing *G*. *duodenalis* under micro-aerobic or entirely anaerobic conditions (through sparging medium) is likely to have a significant impact on transcriptional behaviour, particularly in relation to the oxidative stress response. Therefore, we suggest that the modularity of antioxidant transcription in this protist be further tested under precisely defined atmospheres, together with metabolomic profiling. The consistently high transcription of these pathways during log and stationary phases—when trophozoites are typically harvested for experimentation—is also relevant for contextualizing single time-point studies in these parasites.

Consistent with previous findings that *G*. *duodenalis* exhibits specific stress responses and considerable transcriptional flexibility, the present study indicates that major shifts in transcription in *G*. *duodenalis* might be regulated at least in part through key transcription factors (i.e., MtrR, GntR), and a number of distinct motifs consistent with TF-binding sites, that are enriched in the promoter regions of down- and up-regulated DTGs respectively. The possible role of a two-component-like signal transduction system is particularly interesting and could link cytosolic redox or metabolite sensing and transcriptional changes. This work has been greatly enhanced by the large-scale prediction of putative structures and structural homologs for hypothetical and deprecated proteins, among which were novel antioxidant and signaling proteins among many others. This approach has great potential for illuminating the functions of vast numbers of under-annotated and un-annotated gene products in other important pathogens. The present study also provides a starting point for re-examination of the constituents of the standard trophozoite culture medium, and a reference against which future studies of targeted alterations could be compared. For example, the reaction of dissolved oxygen (dO_2_) with iron may be a source of oxidative stress at early phases during *in vitro* culture of *G*. *duodenalis* trophozoites, and thus concentrations of ammonium ferric citrate and ascorbic acid might need to be reconsidered, particularly as work in other systems has linked ascorbic acid with intracellular iron concentrations [83]. A major finding is that glucose is likely to be in excess in the standard culture media, but its utilization as a carbon source might be limited by the availability of dO_2_, inducing trophozoites to rely on less efficient energy generation pathways in the declining phase. In the future these findings and the detailed longitudinal transcriptomic information presented here, should be used in conjunction with targeted metabolomic investigations in *G*. *duodenalis*, with the aim of creating a completely defined medium. Whereas the present study has used the *G*. *duodenalis* assemblage A genome strain (WB), single time-point profiling of assemblages B [84] and E [85] indicate different transcriptional patterns, which could result from different genomic organization [27,60]. Therefore, similar longitudinal transcriptional investigations of other assemblages are required to better characterize the degree of transcriptional flexibility and the drivers and mediators of transcriptional responses across the species. This work should facilitate a better understanding of the transcriptional flexibility and metabolic preferences of *G*. *duodenalis* under standard culture conditions, and contribute to the development of a completely defined medium for more refined investigations into the biology of this important model eukaryote and pathogen.

## Supporting Information

S1 Table RNA-seq read processing and mapping statistics. (XLSX)

S2 Table Average transcriptional abundance, ranking and fold change for all genes. Differential transcription statistics (FDR <0.05) and putative structural homologs for selected gene products.
(XLSX)

S3 Table Gene Ontology terms in the Biological Process category that are enriched within large differentially transcribed gene groups. (XLSX)

S4 Table KEGG BRITE terms that are enriched within large differentially transcribed gene groups. (XLSX)

S5 Table Average transcriptional abundance for glycolytic enzymes in Fig 4. (XLSX)

S1 Fig Attached, suspended and total *Giardia duodenalis* trophozoite numbers over 96 hours of axenic culture. Flasks were seeded with 10^5^ trophozoites. The decline in total cell number is due to the omission of the pellet of dead and dying cells from the cell count. Error bars represent ± 1 SD, n = 3.
(TIFF)

S2 Fig Total and novel transcript detection as a function of mapped reads for replicate filtered RNA-seq read libraries representing the log, stationary, and declining phases of axenic *Giardia duodenalis* trophozoites. (TIFF)

S3 Fig Distribution of median-normalized transcriptional abundance for all genes at three growth phases. (TIFF)

S4 Fig Transcriptional profiles for ubiquitinylation and proteasomal enzymes. Transcriptional profiles for down-regulated ubiquitinylation enzymes (A); ubiquitinylation enzymes that were not significantly differentially transcribed (B); and proteasome component proteins (C). *significantly differentially transcribed genes encoding proteasome components. Error bars represent ± 1 SEM. UCE: ubiquitin-conjugating enzyme.
(TIFF)

S5 Fig Pearson correlations for transcriptional abundance in the log, stationary and declining growth phases (corresponding to 48, 60 and 96 h; x axis), and transcriptional profiles for WB trophozoites cultured under aerobic (perforated line) and anaerobic conditions (unbroken line) as reported by Ma’ayeh et al. [28]. Correlation between all transcribed genes (A); annotated antioxidant genes (B); annotated glycolytic genes (C); and glycolytic genes encoding proteins involved in glycolysis upstream- (D) and downstream (E) of pyruvate (see Fig 4).
(TIFF)

S6 Fig Transcriptional abundance for variant-specific surface proteins (VSPs) at three growth phases. Gene accession numbers (GL50803) are displayed above the seven most abundant genes.
(TIFF)

S7 Fig Top 20 variant-specific surface proteins (VSPs), ranked by transcriptional abundance for each replicate. (TIFF)

S8 Fig Transcriptional variation relative to mean transcriptional abundance for variant-specific surface proteins (VSPs) at three growth phases. Transcriptional variation between replicates for the same growth phase is expressed as the coefficient of variation (CV; standard deviation ÷ average; y axis). Note the x axis (mean transcriptional abundance) is log. Linear regression lines are displayed.
(TIFF)
