# Supplementary figures and images for: Time-Dependent Transcriptional Changes in Axenic Giardia duodenalis Trophozoites
Source: PLoS Negl Trop Dis. 2015 Dec 4;9(12):e0004261. doi: 10.1371/journal.pntd.0004261 (PMC4670223; doi:10.1371/journal.pntd.0004261)

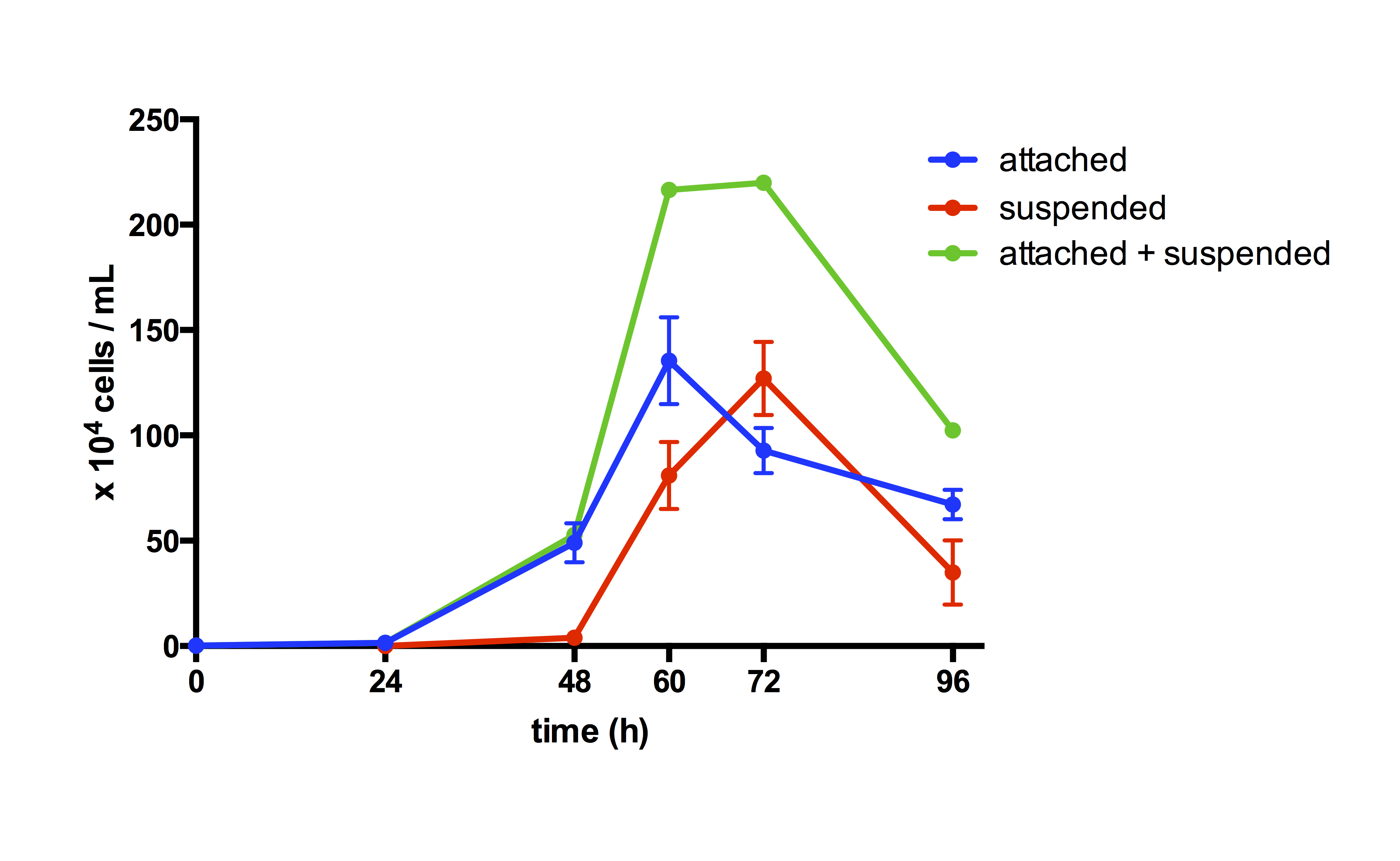

Supplement: S1 Fig — Flasks were seeded with 105 trophozoites. The decline in total cell number is due to the omission of the pellet of dead and dying cells from the cell count. Error bars represent ± 1 SD, n = 3. (TIFF) [file pntd.0004261.s006.tiff]

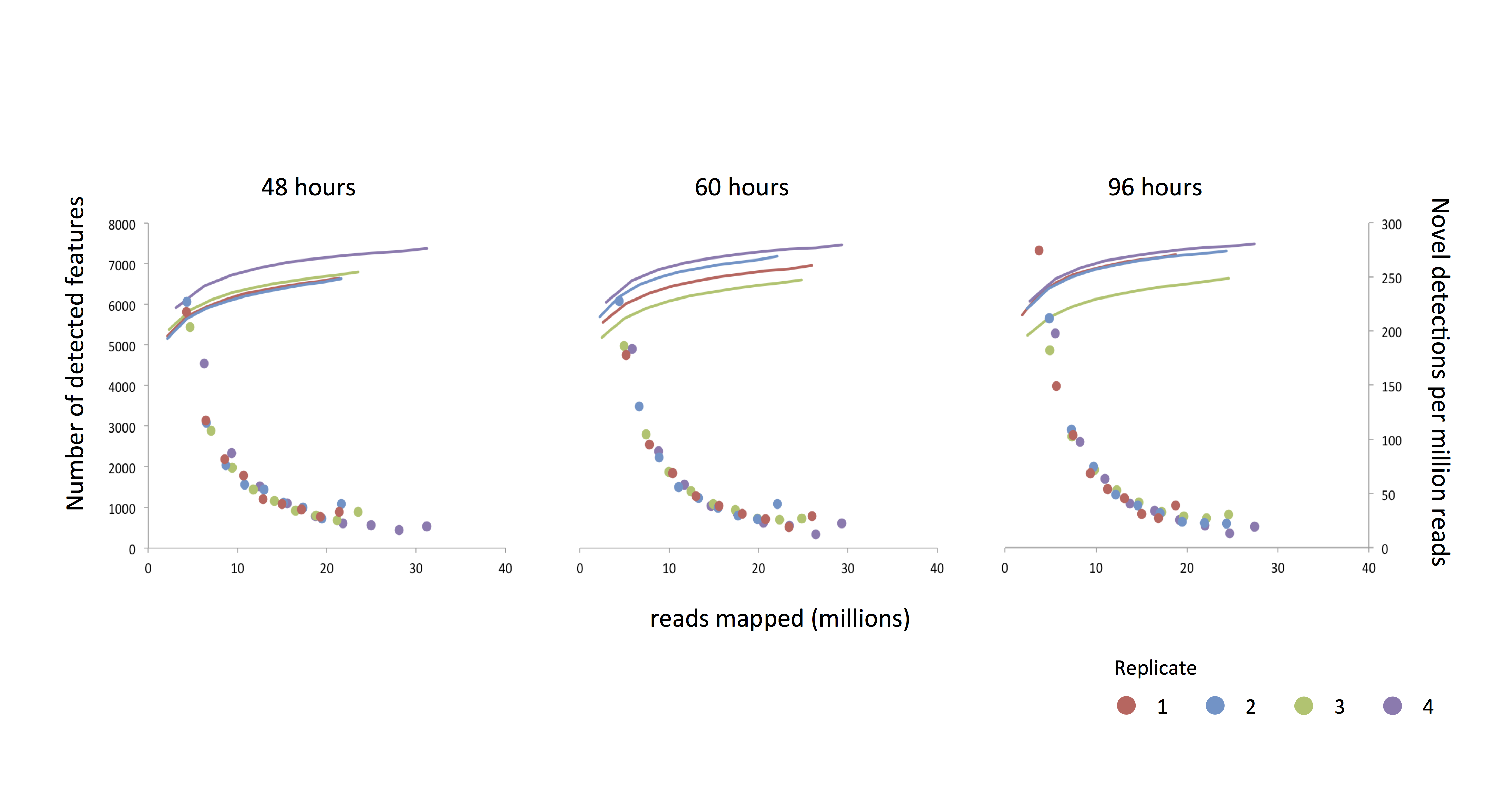

Supplement: S2 Fig — (TIFF) [file pntd.0004261.s007.tiff]

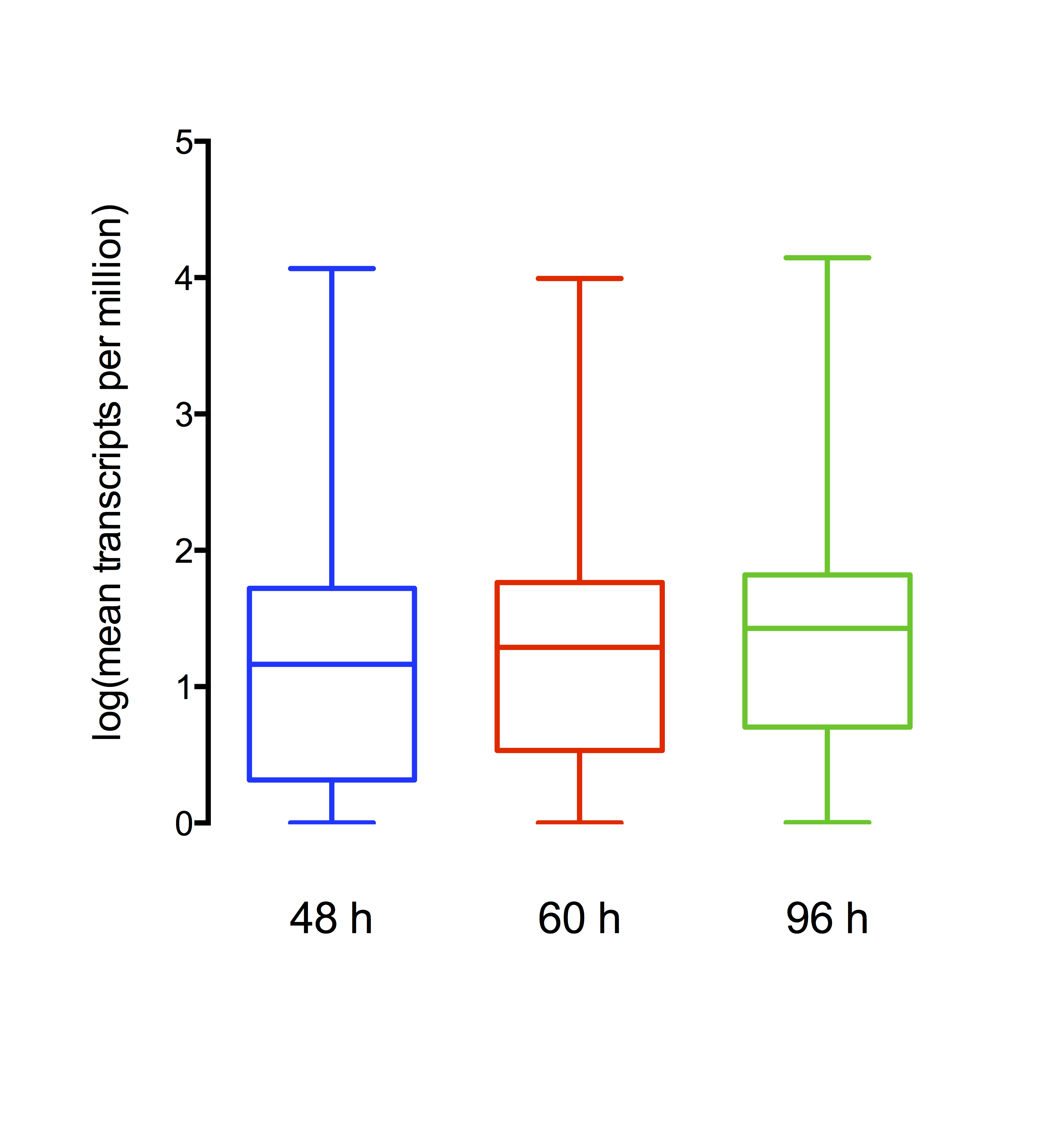

Supplement: S3 Fig — (TIFF) [file pntd.0004261.s008.tiff]

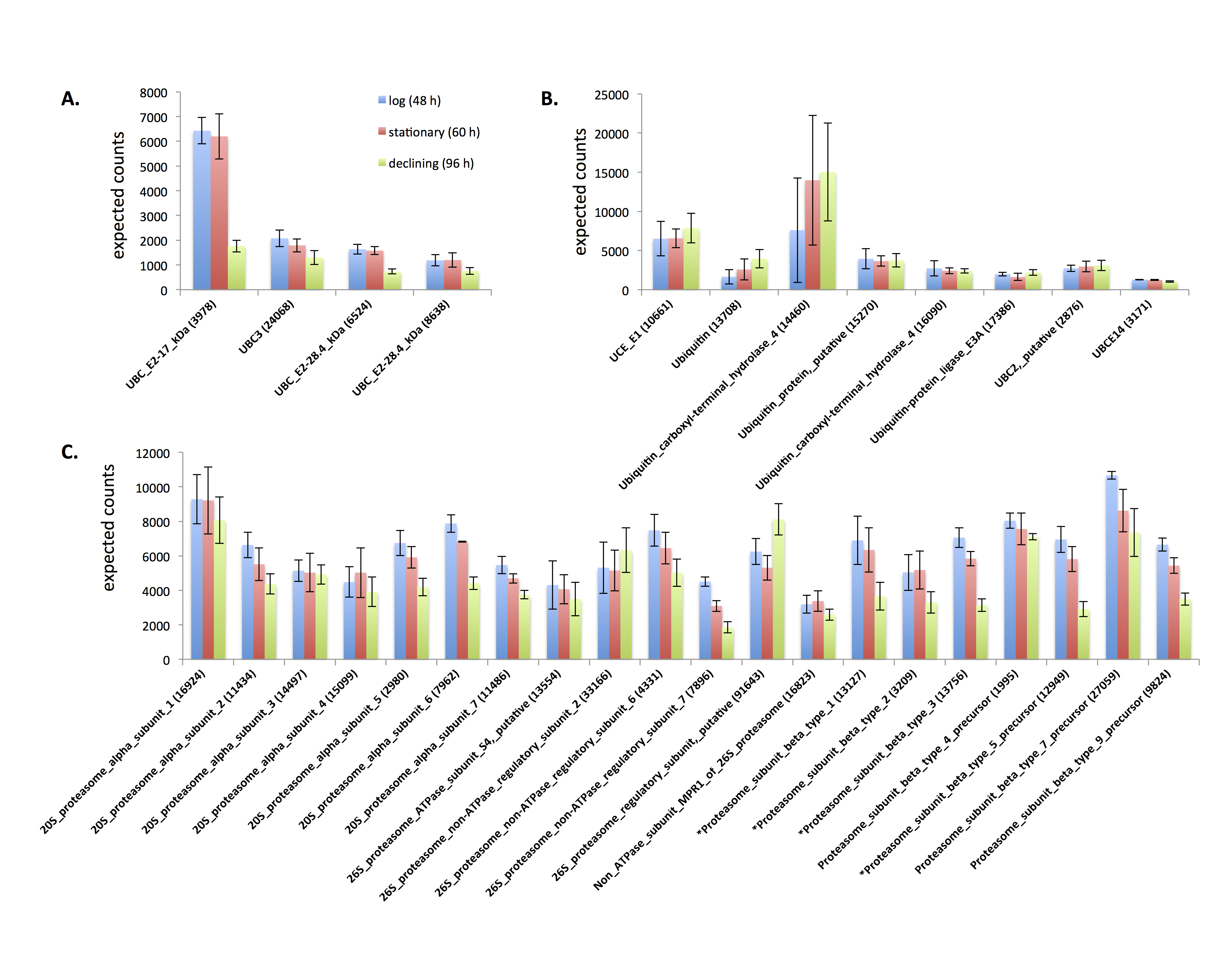

Supplement: S4 Fig — Transcriptional profiles for down-regulated ubiquitinylation enzymes (A); ubiquitinylation enzymes that were not significantly differentially transcribed (B); and proteasome component proteins (C). *significantly differentially transcribed genes encoding proteasome components. Error bars represent ± 1 SEM. UCE: ubiquitin-conjugating enzyme. (TIFF) [file pntd.0004261.s009.tiff]

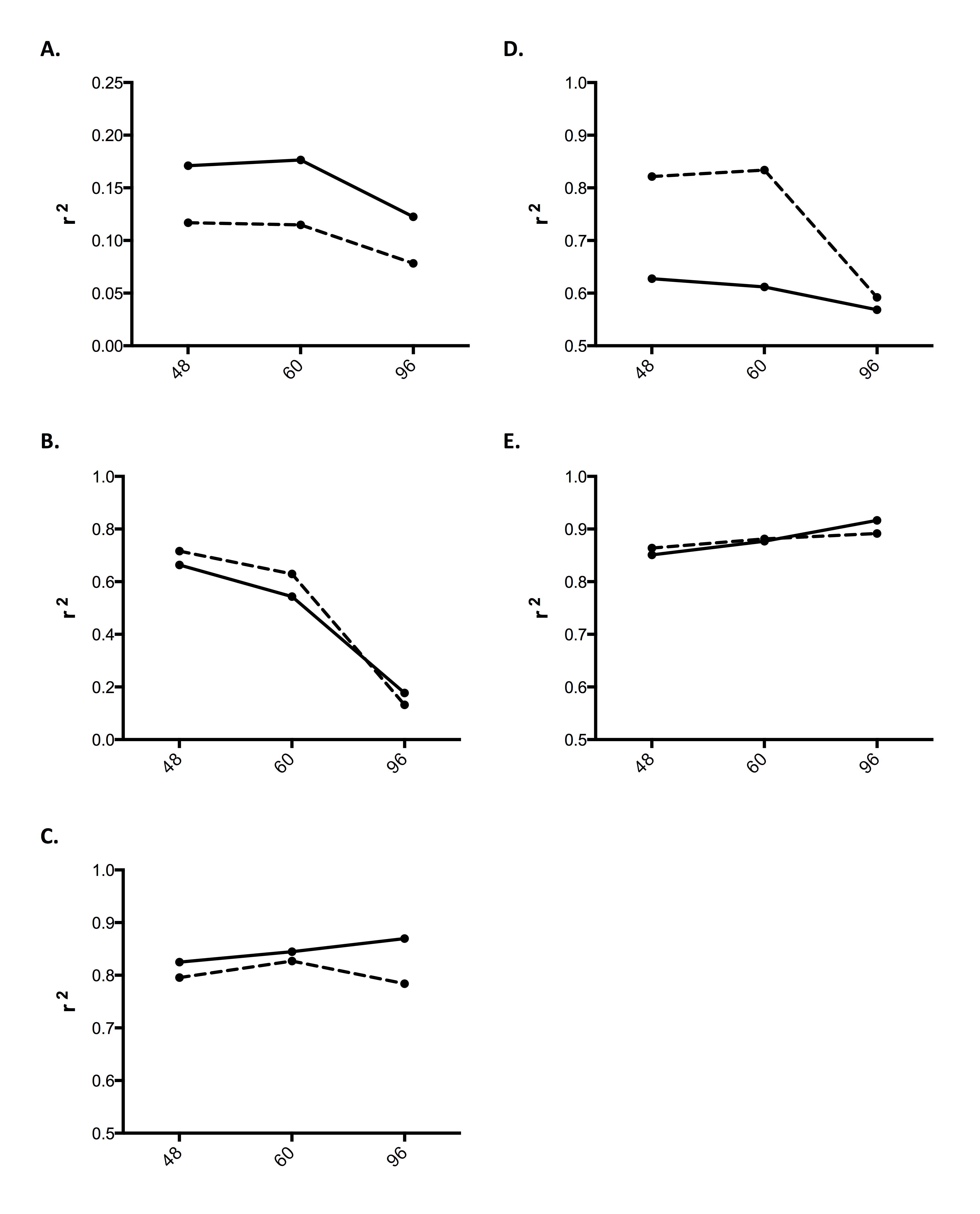

Supplement: S5 Fig — Correlation between all transcribed genes (A); annotated antioxidant genes (B); annotated glycolytic genes (C); and glycolytic genes encoding proteins involved in glycolysis upstream- (D) and downstream (E) of pyruvate (see Fig 4). (TIFF) [file pntd.0004261.s010.tiff]

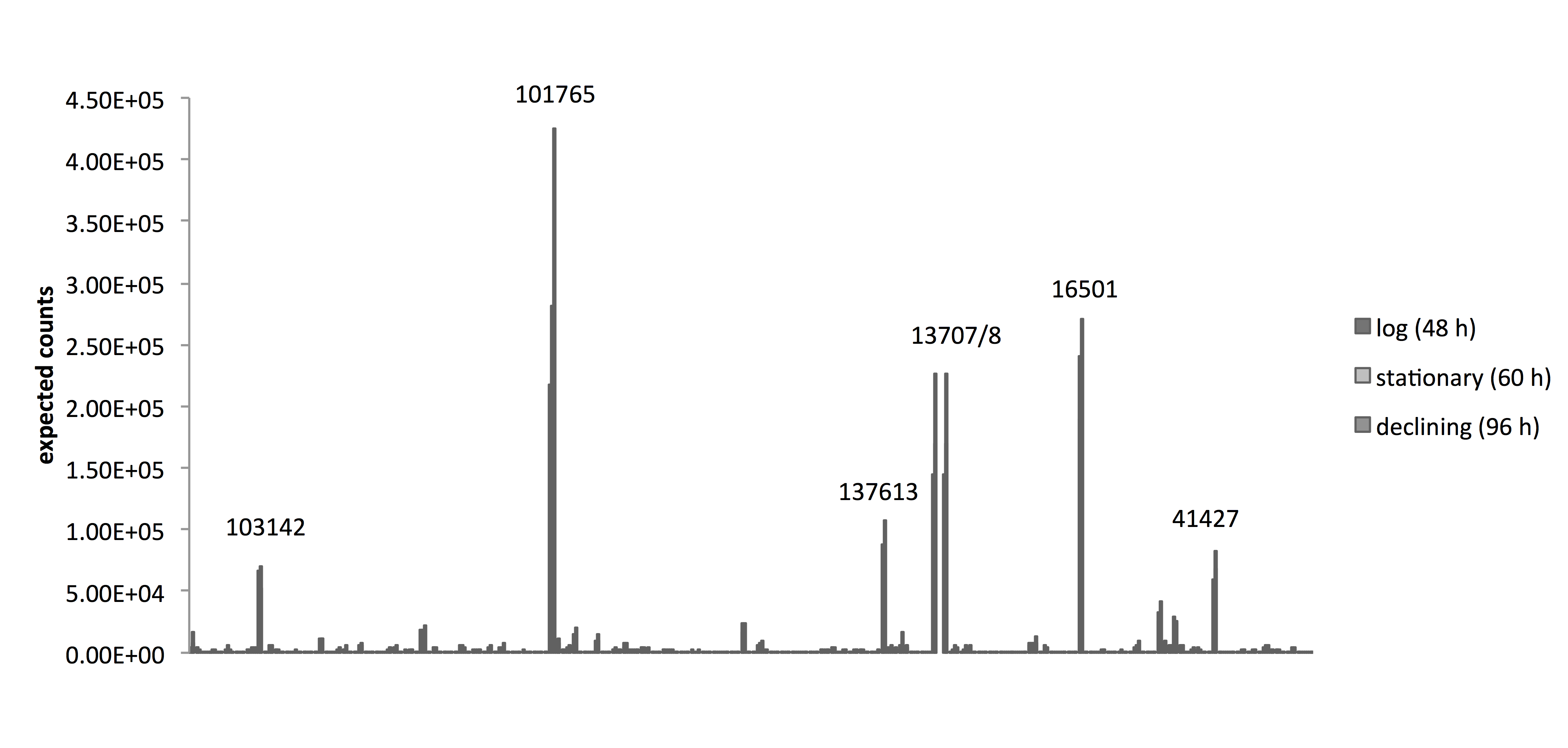

Supplement: S6 Fig — Gene accession numbers (GL50803) are displayed above the seven most abundant genes. (TIFF) [file pntd.0004261.s011.tiff]

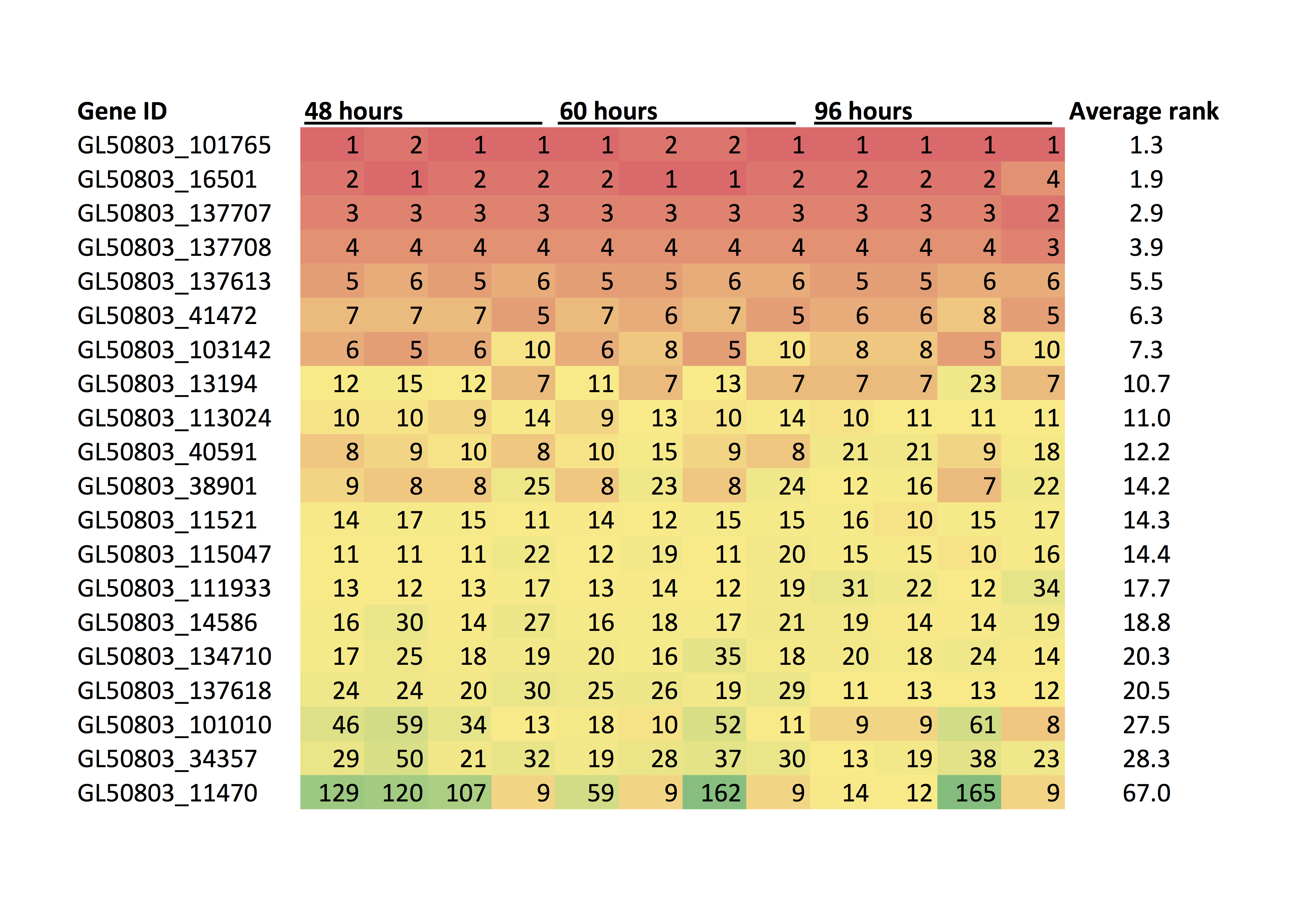

Supplement: S7 Fig — (TIFF) [file pntd.0004261.s012.tiff]

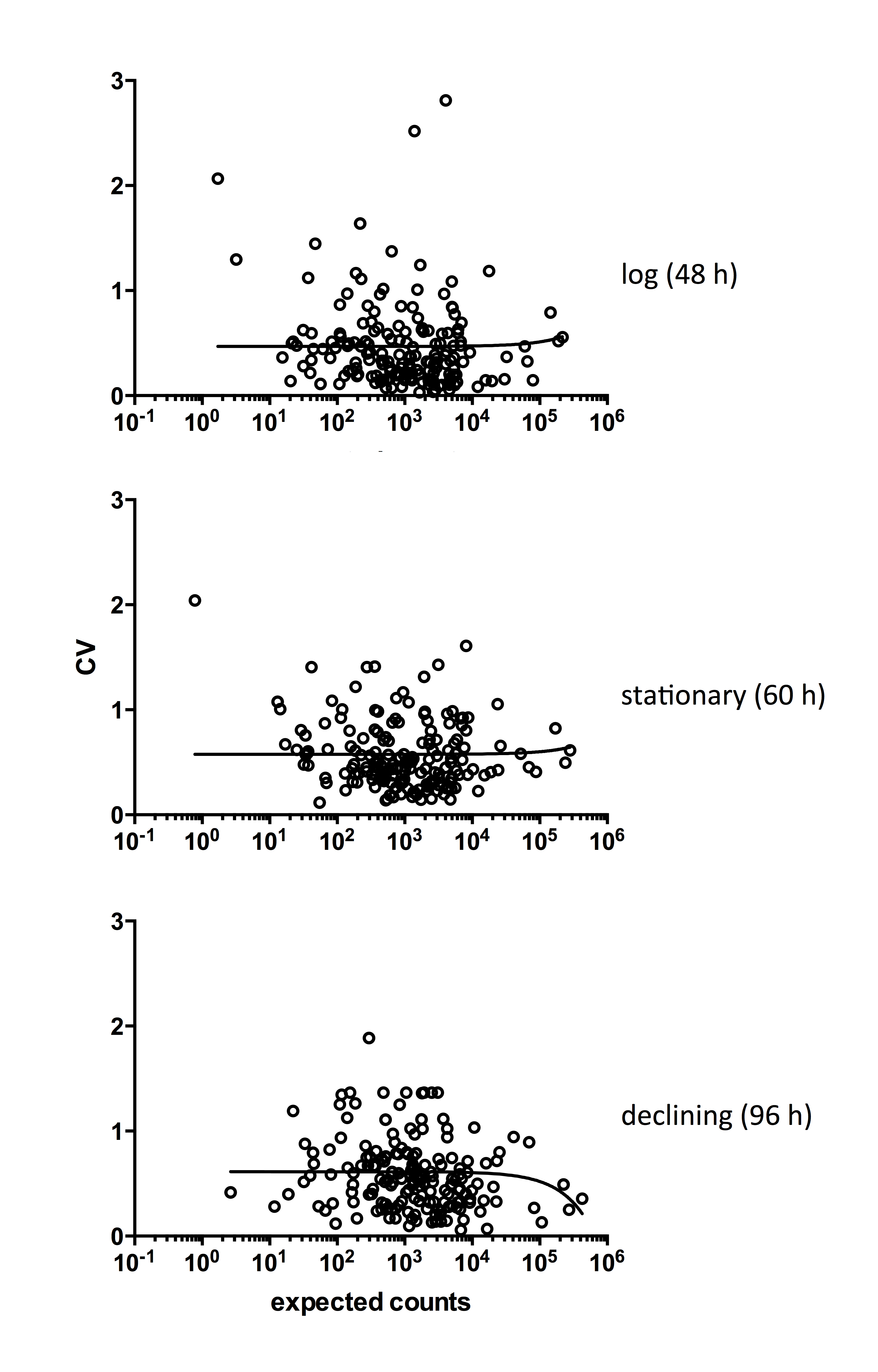

Supplement: S8 Fig — Transcriptional variation between replicates for the same growth phase is expressed as the coefficient of variation (CV; standard deviation ÷ average; y axis). Note the x axis (mean transcriptional abundance) is log. Linear regression lines are displayed. (TIFF) [file pntd.0004261.s013.tiff]
